# Adeno-associated virus serotype 2 induces cell-mediated immune responses directed against multiple epitopes of the capsid protein VP1

**DOI:** 10.1099/vir.0.014175-0

**Published:** 2009-11

**Authors:** Declan Madsen, Emma R. Cantwell, Timothy O'Brien, Patricia A. Johnson, Bernard P. Mahon

**Affiliations:** 1 Cellular Immunology Laboratory, Institute of Immunology, National University of Ireland Maynooth, County Kildare, Ireland; 2 Regenerative Medicine Institute (REMEDI), National University of Ireland Galway, Galway, Ireland; 3 Viral Immunology Laboratory, School of Biotechnology, Dublin City University, Glasnevin, Dublin 9, Ireland

## Abstract

Adeno-associated virus serotype 2 (AAV-2) has been developed as a gene therapy vector. Antibody and cell-mediated immune responses to AAV-2 or AAV-2-transfected cells may confound the therapeutic use of such vectors in clinical practice. In one of the most detailed examinations of AAV-2 immunity in humans to date, cell-mediated and humoral immune responses to AAV-2 were characterized from a panel of healthy blood donors. The extent of AAV-2-specific antibody in humans was determined by examination of circulating AAV-2-specific total IgG levels in plasma from 45 normal donors. Forty-one donors were seropositive and responses were dominated by IgG1 and IgG2 subclasses. Conversely, AAV-2-specific IgG3 levels were consistently low in all donors. Cell-mediated immune recall responses were detectable in nearly half the population studied. *In vitro* restimulation with AAV-2 of peripheral blood mononuclear cell cultures from 16 donors elicited gamma interferon (IFN-*γ*) (ten donors), interleukin-10 (IL-10) (eight donors) and interleukin-13 (IL-13) (four donors) responses. Using a series of overlapping peptides derived from the sequence of the VP1 viral capsid protein, a total of 59 candidate T-cell epitopes were identified. Human leukocyte antigen characterization of donors revealed that the population studied included diverse haplotypes, but that at least 17 epitopes were recognized by multiple donors and could be regarded as immunodominant. These data indicate that robust immunological memory to AAV-2 is established. The diversity of sequences recognized suggests that attempts to modify the AAV-2 capsid, as a strategy to avoid confounding immunity, will not be feasible.

## INTRODUCTION

Adeno-associated virus serotype 2 (AAV-2) is a replication-deficient parvovirus that infects humans, but produces no significant pathology (Blacklow *et al.*, 1968a). The virus consists of a 5 kb single-stranded DNA genome, contained within a non-enveloped icosahedral capsid (Xie *et al.*, 2002). The virus is replication-deficient and requires co-infection with a helper virus such as adenovirus (Ad) or herpes simplex virus (HSV) in order to disseminate (Atchison, 1970; Atchison *et al.*, 1965). Several serotypes of AAV have been investigated for use as gene therapy vectors or recombinant vaccine vehicles (Arruda *et al.*, 2005; High *et al.*, 2004; Kaplitt *et al.*, 2007; Moss *et al.*, 2004, 2007; Yue *et al.*, 2008; Zhang *et al.*, 2003). Although AAV-2 has a relatively small transgene cloning capacity (Kremer & Perricaudet, 1995), a number of features make it an attractive gene therapy vector. The virus has an ability to transduce dividing and non-dividing cells across a range of tissue types (Clark *et al.*, 1997; Flotte *et al.*, 1993; Kaplitt *et al.*, 1994; Koeberl *et al.*, 1997) and is capable of defined integration on human chromosome 19, offering the opportunity for stable, controlled transgene expression (Kotin *et al.*, 1990). Finally, AAV-2 is assumed to induce minimal host immune responses, perhaps due to its helper virus dependence. The modest immune responses reported to date have suggested a reduced risk of vector or transduced cell destruction following administration (Zaiss *et al.*, 2002; Zaiss & Muruve, 2005).

The innate immune response to AAV-2 appears to be weak and transient in animal models (Samulski & Giles, 2005; Zaiss *et al.*, 2002). However, little is known about the immune response to AAV-2 infection in humans, beyond the prevalence of infection (Blacklow *et al.*, 1968a, b; Gao *et al.*, 2004). Observation of the adaptive response to AAV-2 in animal models has demonstrated robust primary responses, including specific neutralizing antibody (Halbert *et al.*, 1997; Xiao *et al.*, 1999, 2000). The human antibody response to AAV-2 has been characterized, but seroprevalence varies between 30 and 96 % depending on the population sampled (Chirmule *et al.*, 1999; Erles *et al.*, 1999; Halbert *et al.*, 2006). This broad range might reflect the lack of standardized methods for assessing AAV-2 serology. AAV-2 infection is thought to have occurred in at least 20 % of humans before the age of 10 years (Erles *et al.*, 1999). The detection of AAV DNA in amniotic fluid also suggests that the virus may be present at birth in many humans, possibly due to reactivated latent infection in the mother during pregnancy (Tobiasch *et al.*, 1994).

The cell-mediated adaptive immune response to AAV has received little attention and there has been some assumption that AAV does not provoke a significant response (Büning *et al.*, 2003; Hernandez *et al.*, 1999; Samulski & Giles, 2005; Zaiss & Muruve, 2005). The detection of AAV-2-specific IgM (Erles *et al.*, 1999) might indicate that AAV-2 behaves like a T-cell-independent antigen. However, there is evidence of notable cell-mediated responses to AAV-2 from a recent gene therapy trial which reported declining transgene expression and indications of tissue damage, with concurrent cytotoxic T-cell responses (Manno *et al.*, 2006). Mouse models have since been used to characterize T-cell responses to AAV-2 in greater detail (Chen *et al.*, 2006; Li *et al.*, 2007; Wang *et al.*, 2007). However, inbred mouse strains display limited MHC diversity in comparison with the highly polymorphic human leukocyte antigen (HLA) genes, and cell-mediated immunity in mice may not reflect immunity in human populations.

Based on the emerging data on cell-mediated immunity in humans and animal models, it is clear that the cell-mediated response to AAV-2 needs further characterization in the natural host if the virus is to be used confidently in a clinical setting. Thus, to assess the feasibility of repeated use of AAV-2 vectors in human populations, the immune response to AAV-2 was characterized from a panel of 45 human blood donors. A high seroprevalence was observed and this was further characterized by examination of the immunoglobulin subclasses evoked by natural infection. For the first time, to our knowledge, IgG responses were correlated with proliferative cell-mediated responses from the same donors. Furthermore, these were compared with HLA haplotype and an analysis of the profile of cytokine responses induced by AAV-2 stimulation. Finally, a large number of T-cell epitopes were identified on the AAV-2 virus capsid. This study is, to our knowledge, the most complete characterization of the human immune response against AAV-2 to date, examining both arms of the adaptive response. The diversity of stimulatory targets identified on the AAV-2 capsid and the prevalence of the cell-mediated immune response detected undermine the notion that the AAV-2 capsid might easily be modified to avoid cell-mediated immunity.

## METHODS

### Human peripheral blood mononuclear cell (PBMC) and blood plasma donations.

Human whole blood was provided by 45 anonymous Irish healthy donors. PBMCs were isolated by density-gradient centrifugation (Lymphoprep; Axis-Shield) as described previously (Ryan *et al.*, 1998). Isolated PBMCs were resuspended in 8 % (v/v) heat-inactivated FCS/RPMI 1640 medium (cRPMI) (Invitrogen). Blood plasma was isolated from whole blood by centrifugation at 10 000 ***g*** for 10 min. Human material was acquired and used in accordance with approval from the local Research Ethics Committee [National University of Ireland (NUI) Maynooth].

### Virus preparation.

AAV-2 for immunological assays was provided by the Regenerative Medicine Institute (REMEDI; NUI, Galway) and consisted of an unmodified AAV-2 capsid encasing a modified AAV-2 genome encoding a reporter gene, as previously described (Stender *et al.*, 2007). Virus was purified using an iodixanol density gradient followed by elution through a heparin column. Titres were determined by real-time PCR following DNase1 and proteinase K degradation. Concentrations were expressed as DNase-resistant particles per μl (d.r.p. μl^−1^). Virus preparations were resuspended in sterile buffer (PBS, 1 mM MgCl_2_, 2.5 mM KCl) and stored at −80 °C. Reagents for vector preparation were sterilized prior to use.

### Virus capsid peptides.

The full VP1 sequence incorporates the VP2 and VP3 sequences of the AAV-2 capsid (Xie *et al.*, 2002). A panel of 91 20-mer peptides were synthesized mostly with a 12-mer overlap to adjacent sequences (Supplementary Table S1, available in JGV Online). Peptides were synthesized commercially (Mimotopes) and provided lyophilized. These were reconstituted in 0.1 % (v/v) acetic acid at a concentration of 25 mg ml^−1^, split into aliquots and stored at −80 °C. Before use, peptides were diluted in PBS to a working concentration of 1 mg ml^−1^.

### AAV-2-specific total IgG ELISA.

Total IgG levels were determined in plasma from donors using an optimized in-house assay. Ninety-six-well microassay plates (Nunc) were coated with AAV-2 at 1×10^9^ d.r.p. ml^−1^ in carbonate coating buffer (150 mM Na_2_CO_3_, 350 mM NaHCO_3_, pH 9.6) for 12 h at 4 °C. Plates were blocked using 5 % (w/v) sucrose, 1 % (w/v) BSA, 0.05 % (v/v) Tween 20 in PBS. Blood plasma was diluted in 1 % (w/v) skimmed milk powder in PBS and incubated at 37 °C for 2 h before detection of human IgG using a biotinylated anti-human IgG (Sigma-Aldrich). Detection was performed using a streptavidin–horseradish peroxidase conjugate and tetramethyl benzidine substrate. Five washes were performed between each step. In the absence of AAV-2-specific IgG international standards, two donors (22 and 41), previously characterized as reference donors, were selected as representative seronegative and seropositive samples. These samples were included in all assays to allow inter-assay comparison. AAV-2-specific IgG data for the negative reference sample were assigned an AAV-2 antibody titre of 1 unit, whilst the reference seropositive donor was assigned an AAV-2 antibody titre of 10 units. This allowed quality control and normalization of results for meaningful comparison of data between assays. Samples were considered seropositive if they scored above the seronegative cut-off plus two standard deviations.

### AAV-2-specific IgG subclass ELISA.

IgG subclass concentrations were determined in plasma samples from 41 AAV-2-IgG-seropositive blood donors. Assays were performed in an adaptation of the protocol for total IgG detection. Plates were probed for human IgG using anti-human IgG subclass-biotin antibodies for subclasses IgG1–IgG4 (Merck). Detection and washing were performed as for the total IgG. To validate these assays in the absence of international reference standards, a series of verification assays were performed using the above detection reagents to detect reference sera with standard concentrations of purified human IgG 1, 2, 3 or 4 (Nordic Laboratories) directly coated to the assay plate. In addition, the detection reagents for IgG1–4 were validated against a similar but unrelated virus (parvovirus B19) prevalent in this population. Representative data for IgG3 are given in Supplementary Fig. S1 (available in JGV Online).

### Proliferation assay.

Proliferation in response to AAV-2 restimulation was determined from PBMC cultures isolated from 41 healthy Irish blood donors. Only viable, non-apoptotic PBMC populations were used. Human PBMC (1×10^6^ cells ml^−1^) were cultured in triplicate with AAV-2 (1×10^10^ d.r.p. μl^−1^) or VP1 peptide (40 μg ml^−1^), cRPMI alone (negative control) or with concanavalin A (Con A) (5 μg ml^−1^) (positive control). Cultures were incubated at 37 °C, 5 % CO_2_ for 96 h. After 96 h, 100 μl supernatant was removed from each well and frozen at −20 °C for cytokine analysis. Culture media were then replaced with cRPMI containing ^3^H-thymidine (92.5 μBq ml^−1^) and incubated for 5 h, before detection of radioactive incorporation by scintillation counting (Ryan *et al.*, 2007). Results (c.p.m.) were expressed as stimulation indices (SI), calculated as the fold proliferation increase over the negative control (Corcoran *et al.*, 2000). For assays employing VP1 peptides, SI values were considered positive if greater than one standard deviation above the mean SI for stimulated wells.

### Definition of T-cell epitopes.

Each VP1 peptide sequence used (Supplementary Table S1) possessed a 12-mer overlap with one upstream and one downstream sequence (except for the terminal peptides), so it was likely that any given epitope would be present in two adjacent peptides. Only responses where two or more adjacent peptides supported positive proliferation were considered to represent a valid epitope. In parallel, a bioinformatics approach was used to predict HLA class I- and class II-restricted epitopes within the AAV-2 VP1 capsid sequence (SYFPEITHI epitope prediction algorithm) (Rammensee *et al.*, 1999). Predicted sequence output sizes were set at 9-mer for class I epitopes and 15-mer for class II epitopes. Due to the large number of sequences generated for each HLA allele, only epitopes with a score of >20 were used for comparison with sequences detected *in vitro*.

### Detection of cytokines.

Cytokine levels were determined in supernatants from 16 PBMC cultures representing samples that supported proliferation in response to AAV-2 stimulation, and where sample abundance allowed analysis. Commercial human gamma interferon (IFN-*γ*) and human interleukin (IL)-10 ELISA kits (Immunotools) or matched antibodies for human IL-13 (R&D Systems) were used for cytokine detection, according to the manufacturer's instructions, except for IL-13, where antibody was diluted in carbonate coating buffer to 4 μg ml^−1^. Cytokine concentrations were determined by comparison with reference standards of known concentration.

### Characterization of HLA haplotypes.

HLA haplotypes were characterized for 16 PBMC donors supporting *in vitro* proliferative responses to AAV-2 stimulation described above. Sequence-specific primer (SSP) PCR for HLA A, B, C, DR and DQ were performed. DNA was isolated from PBMC using a Generation Capture Column kit (Qiagen) according to the manufacturer's protocol. HLA A, B, C, DR and DQ were characterized using an SSP-based PCR kit (Texas BioGene) in a split 96-well tray format. Amplified samples were resolved on a 2 % agarose gel and analysed using SSPal HLA analysis software (Texas BioGene) following the manufacturer's protocol.

## RESULTS

### AAV-2-specific IgG1 and IgG2 are prevalent in a population of Irish blood donors

**Fig. 1. f1:**
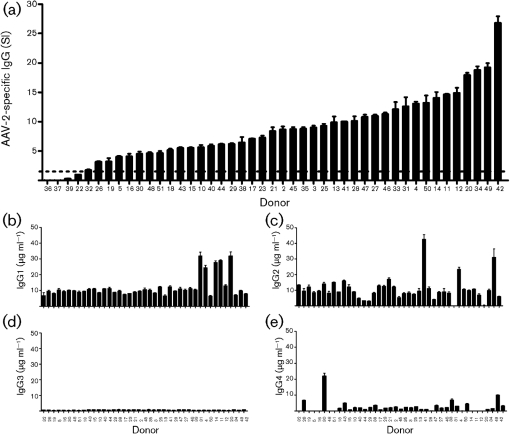
Serological response to AAV-2 in Irish blood donors. (a) AAV-2-specific total IgG in human donor plasma (*n*=45). Specific IgG was determined by indirect AAV-2 antigen ELISA, normalized by comparison with standard positive and negative samples. The negative cut-off (dashed line) was defined at 2 sd above the concentration of a known seronegative standard. (b–e) AAV-2-specific IgG subclasses present in IgG-positive samples (*n*=41). IgG1(b), IgG2 (c), IgG3 (d) and IgG4 (e) determined by AAV-2-specific direct antigen ELISA for triplicate samples and quantified by comparison with known reference sera.

The reported seroprevalence of AAV-2-specific antibody is highly variable (Chirmule *et al.*, 1999; Erles *et al.*, 1999; Murphy *et al.*, 2009). Therefore, the seroprevalence of AAV-2-specific total IgG was examined in a population of healthy Irish volunteer blood donors. Plasma from 45 donors was assayed for AAV-2-specific IgG by indirect ELISA using in-house reference sera as controls. Donors were considered AAV-2-seropositive if the IgG level was two standard deviations greater than a known seronegative sample. Forty-one of the 45 donors assayed displayed AAV-2-specific IgG above this cut-off and were therefore described as seropositive (Fig. 1a[Fig f1]). Using this approach, the mean titre of AAV-2-specific IgG observed in seropositive donors was 9.4±5.2 units.

Whilst total IgG gives an indicator of virus exposure in a population, it does little to inform understanding of the immunological mechanisms in operation. Therefore, plasma samples from IgG-seropositive donors were further examined to determine the IgG subclasses involved in the specific recognition of AAV-2 (Fig. 1b–e[Fig f1]). Significant levels of AAV-2 specific IgG1 were detected in all IgG-positive samples assayed (*P*≤0.05), with a mean concentration of 11.9±6.7 μg ml^−1^ (Fig. 1b[Fig f1]). AAV-2-specific IgG2 was also prevalent and detected in more than 95 % of samples at a similar mean concentration (10.8±7.5 μg ml^−1^) (Fig. 1c[Fig f1]). Interestingly, although AAV-2-specific IgG3 was detected in this donor population, it was uniformly weak (mean concentration 0.7±0.2 μg ml^−1^) (Fig. 1d[Fig f1]) despite strong IgG3 responses to other unrelated antigens (Supplementary Fig. S1). Concentrations of the AAV-2-specific IgG4 were variable (mean 2.5±3.8 μg ml^−1^) (Fig. 1e[Fig f1]). Twelve donors showed no detectable AAV-2-specific IgG4 and only four of the samples (donors 26, 30, 33 and 49) displayed IgG4 concentrations greater than one standard deviation above the mean.

### AAV-2 induces a recall cell-mediated response

**Fig. 2. f2:**
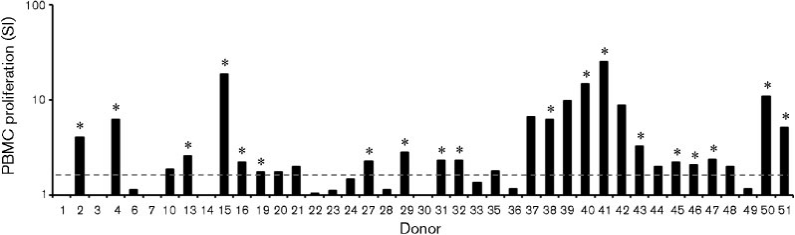
AAV-2-stimulated human PBMC proliferation *in vitro*. Proliferation (detectable in 19 of 41 cultures) was determined by titrated thymidine incorporation for triplicate samples and expressed as SI. Responses were only considered positive (*) if they supported both a mean SI ≥1.5 (dashed line) with a significance of *P*≤0.05 (paired *t*-test).

It has been speculated that AAVs do not elicit significant cell-mediated immune responses (Büning *et al.*, 2003; Hernandez *et al.*, 1999; Samulski & Giles, 2005); however, the detection of a class-switched AAV-2-specific IgG response suggested that T-cell help had been evoked by AAV-2 exposure in the study population. Therefore, the cell-mediated immune response to AAV-2 was examined. PBMC were isolated from the same panel of seropositive donors described above (*n*=41). These cultures were stimulated *in vitro* with AAV-2 and assessed for their capacity to support AAV-2-specific proliferation. PBMC from 19 of 41 Irish blood donors sampled displayed significant proliferation in response to restimulation (Fig. 2). It was therefore clear that AAV-2 induced memory responses sufficient to support a recall response to exogenous antigen in a considerable number of donors.

**Fig. 3. f3:**
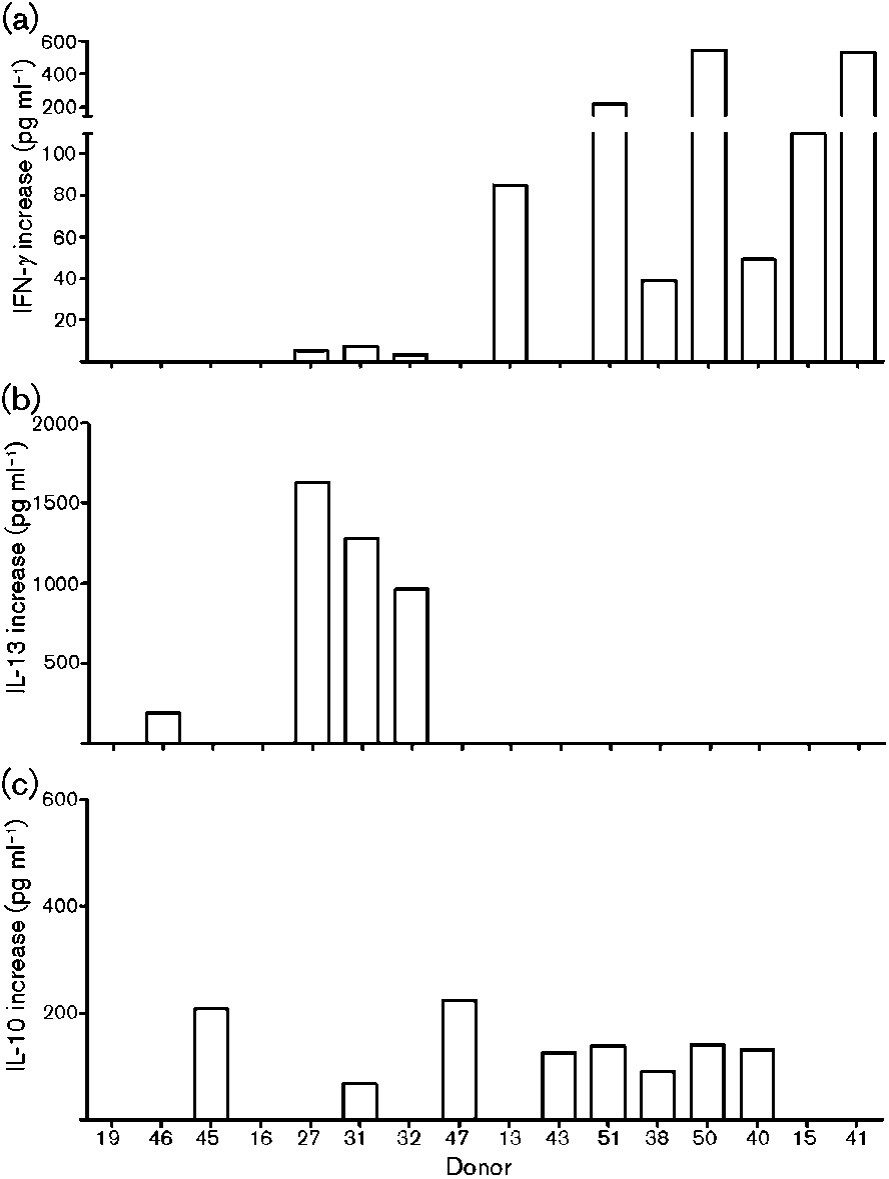
Cytokine responses to AAV-2-stimulated human PBMCs *in vitro*. Production of IFN-*γ* (a), IL-13 (b) or IL-10 (c) by PBMC cultures (*n*=16, proliferation-positive in Fig. 2) stimulated with AAV-2. Results are expressed as the mean of triplicate determinations.

To characterize the cellular response to AAV-2 further, supernatants from antigen-stimulated cultures (*n*=16) were assessed for the production of IFN-*γ*, IL-10 and IL-13, cytokines characteristic of polarized CD4^+^ T helper cell recall responses. Ten cultures (donors 13, 15, 27, 31, 32, 38, 40, 41, 50 and 51) produced significantly increased IFN-*γ* (*P*≤0.05) (Fig. 3a[Fig f3] and Table 1). Cultures from four donors (27, 31, 32 and 46) showed increased IL-13 (Fig. 3b[Fig f3]); whereas increased IL-10 was present in eight cultures (donors 31, 38, 40, 43, 45, 47, 50 and 51; mean concentration 140 pg ml^−1^) (Fig. 3c[Fig f3], Table 1). Although no clear polarization of cytokine responses was seen in this population, the detection of IFN-*γ*, IL-10 or IL-13 in recall responses suggested that long-lived CD4^+^ T-cell responses were evoked by AAV-2 in at least some of the study population.

### Antigenic sequences on AAV-2 recognized by T cells

**Fig. 4. f4:**
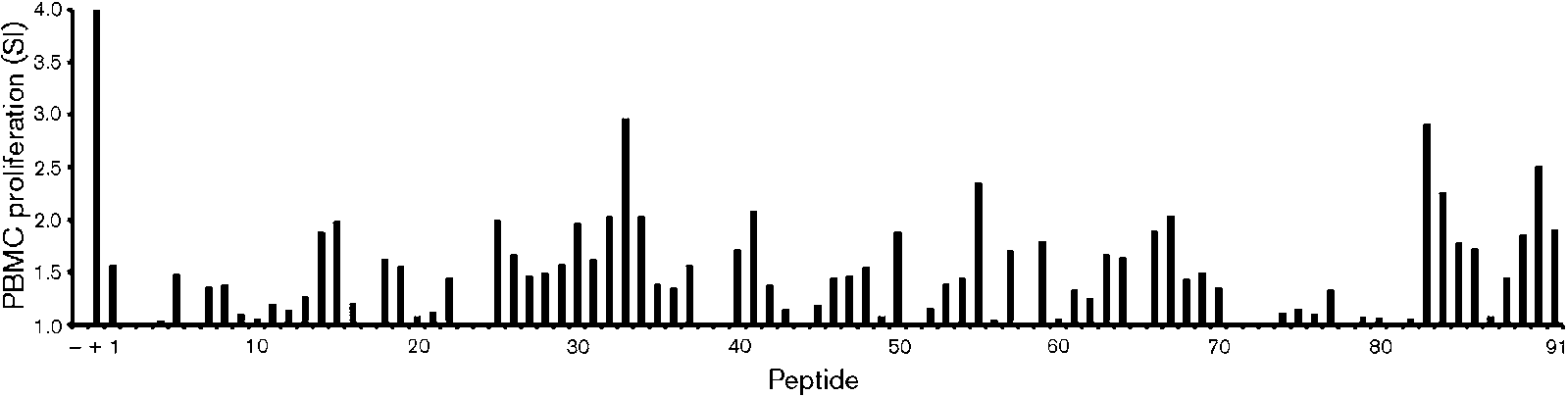
Human PBMC proliferation in response to peptides corresponding to AAV-2 VP1. PBMCs (representative donor 51 shown here) were cultured in the absence (−, negative control) or presence (+, positive control) of 40 μg peptide or mitogen ml^−1^. Peptide sequences (numbered 1–91) are given in Supplementary Table S1. Proliferation of greater than one sd above the mean was considered positive. Results are expressed as the mean of triplicate determinations.

There has been limited examination of the targets of cell-mediated immunity to AAV-2 in humans. Therefore the ability of synthetic peptides corresponding to AAV-2 VP1 capsid peptides to support recall responses from human PBMCs was examined. PBMCs from the 16 donors characterized in Fig. 4 (donors 13, 15, 16, 19, 27, 31, 32, 38, 40, 41, 43, 45, 46, 47, 50 and 51) were stimulated with overlapping 20-mer peptides corresponding to the complete protein sequence of VP1. The sequence for VP1 contains the full sequence of the alternative capsid proteins VP2 and VP3, thus stimulation of PBMCs with overlapping peptides from VP1 allowed identification of stimulatory epitopes across the entire AAV-2 capsid sequence. Cultures were assessed for proliferation in response to these antigens (Fig. 4). A stringent definition of an epitope was chosen; only stimulating pairs of adjacent 20-mer sequences from more than one donor were considered to represent an antigenic sequence recognized by T cells, with the 12-mer consensus sequence overlap assumed to contain the T-cell epitope. A total of 17 consensus sequences were identified that were recognized by two or more donors. These 17 common antigenic sequences (Table 2, labelled epitopes A–Q) were identified across a total of six donors. All of the sequences identified showed greater than 85 % homology with high-scored HLA class I- and class II-restricted VP1 epitopes predicted by bioinformatics. A further 42 unique candidate epitopes were identified that were recognized by single donors only (Supplementary Table S2, available in JGV Online).

### AAV-2 T-cell epitopes on AAV-2 VP1 may be presented by multiple HLA haplotypes

T-cell recognition of antigens in mammals is MHC restricted. Humans are an outbred population with highly polymorphic HLA profiles. In order to establish if the AAV-2 epitopes identified were associated with particular haplotypes, the HLA haplotype for each AAV-2 respondent donor was determined by SSP PCR for the HLA-A, -B, -C, DRB and DQB loci (Tables 3 and 4). As expected, the donors characterized displayed considerable haplotype diversity. No obvious correlations between HLA expression and epitope recognition were detectable in a population of this size; however, the key observation from this study was that donors of different HLA haplotypes (Tables 3 and 4) recognized at least 16 epitopes, indicating that certain regions of the AAV-2 capsid are recognized promiscuously and may be reasonably considered immunodominant.

## DISCUSSION

The paucity of data available on immune responses to AAV-2 raises the possibility that immunity might complicate the use of AAV-2 as a therapeutic vector. In this study, AAV-2-specific IgG was present in plasma from 41 of 45 donors sampled (>90 %). IgG1 and IgG2 were the predominant AAV-2-specific subclasses present. IgG3 levels were limited and IgG4 was variable and often absent entirely. Human T-cell proliferation in response to whole AAV-2 stimulation was demonstrated in nearly half (19 of 41) of PBMC cultures studied. The cytokine profiles associated with these responses were diverse, but IFN-*γ* and IL-13 production were detected. Fifty-nine candidate T-cell epitopes were identified within the VP1 capsid sequence. Seventeen epitopes were identified on the VP1 protein of AAV-2 which were recognized by more than one donor; no significant correlation between stimulating epitope and respondent donor HLA haplotype was observed, suggesting that these represent promiscuously recognized immunodominant epitopes. This study, to our knowledge, represents the most detailed combined examination of cell-mediated and humoral immunity to AAV-2 in humans to date. This study demonstrates that both humoral and cell-mediated memory for AAV-2 is prevalent in the Irish population, supporting the hypothesis that immunity will complicate the use of AAV-2 in therapy. Capsid modification strategies are unlikely to be a practical solution due to the variety of epitopes recognized; however, screening for patient cell-mediated and humoral responses may be an invaluable tool in bringing effective AAV-2 vectors to clinical use.

Given the known prevalence of AAV-2 infection in humans (Chirmule *et al.*, 1999; Erles *et al.*, 1999; Halbert *et al.*, 2006), it is conceivable that widespread humoral memory for the virus might negatively affect the usefulness of the virus as a gene therapy vector. In this study, 41 of 45 donors studied displayed significant titres of AAV-2-specific IgG in blood plasma (Fig. 1). Although this is the largest study to examine both humoral and cell-mediated responses to AAV-2 in the same population, a sample of 45 donors is still small and larger studies in populations with greater genetic diversity would be beneficial. Nevertheless, a seroprevalence greater than 90 % is high when compared with AAV-2 seroprevalence data obtained in other European studies but similar when compared with populations from the USA (Chirmule *et al.*, 1999). As no AAV-2-specific IgG standards exist and as there is no standardized method for assaying AAV-2 antibody, it is likely that the variability observed between studies is as much a result of differing methods as it is of geographical differences and population demographics. An urgent need exists to develop reference standards for AAV serology; currently it is difficult to compare titres between studies in a quantitative way.

In this study, AAV-2-specific IgG consisted primarily of IgG1 and IgG2, with low levels of IgG3 and variable levels of IgG4 present in all donors (Fig. 1). The presence of IgG1 (Fig. 1b[Fig f1]) was expected, as this subclass is commonly induced following viral infections such as measles, hepatitis B, human T-lymphotrophic virus type 1 (HTLV-1) and rubella (Franssila *et al.*, 1996; Gregorek *et al.*, 2000; Lal *et al.*, 1993; Thomas & Morgan-Capner, 1988; Toptygina *et al.*, 2005). IgG1 is also induced by B19V which, like AAV-2, is a member of the parvoviridae (Franssila *et al.*, 1996). IgG2 was also a major constituent of the AAV-2-specific antibody response, comprising an average 42 % of total IgG (Fig. 1c[Fig f1]). The proportions of AAV-2-specific IgG1 and IgG2 detected in this study broadly agree with data recently reported by Murphy *et al.* (2009). Whilst IgG2 is a component of serological responses to measles and HTLV-1, it is notable that it is not a significant component of the response to the parvovirus B19V (Franssila *et al.*, 1996; Lal *et al.*, 1993; Toptygina *et al.*, 2005). This suggests that there are differences between how the immune response develops against B19V and AAV-2, perhaps reflecting the requirement of AAV for a helper virus to disseminate.

The IgG3 subclass is usually a significant component of virus-induced IgG, typically comprising 12–50 % of circulating virus-specific IgG for measles, rubella, HTLV-1 and hepatitis B in seropositive individuals (Gregorek *et al.*, 2000; Kalvenes *et al.*, 1996; Lal *et al.*, 1993; Toptygina *et al.*, 2005). Although AAV-2-specific IgG3 was detected in all seropositive donors characterized, this subclass constituted an average of just 2.6 % (0.7±0.2 μg ml^−1^) of the total AAV-2-specific IgG detected (Fig. 1d[Fig f1] and Table 1). Given the robust humoral response induced by AAV-2, low levels of IgG3 are surprising but supported by observation of similar levels from a recent study from the USA (Murphy *et al.*, 2009). The average concentration of virus-specific IgG3 (0.6 μg ml^−1^) observed in that study was similar to our observations; however, we did not observe any case where IgG3 represented more than 6 % of total AAV-2-specific IgG from any donor examined (Table 1). The difference between the results reported by Murphy *et al*. (2009) and those reported here may reflect differences in population genetic background, or the prevalence and nature of various helper virus infections.

The low seroprevalence of IgG3 against AAV-2 deserves further attention. Recognition of a particulate antigen in the inductive immune sites of the upper respiratory tract typically produces a higher proportion of IgG3-producing B cells than recognition in the circulatory system (Jefferis & Kumararatne, 1990). Given that the route of natural AAV-2 infection is oral/respiratory tract (Blacklow *et al.*, 1968a; Gould & Favorov, 2003; Rabinowitz & Samulski, 2000), the failure of AAV-2 to induce significant IgG3 in any donor examined here is unexpected. It might be that AAV-2 has some capacity to evade or subvert strong IgG3 induction, which would represent a means of evading the complement cascade. This hypothesis is supported by the observation that the classical complement pathway is only induced weakly by high titres of AAV-2 and the alternative complement pathway is not activated (Zaiss *et al.*, 2008). As IgG3 is the primary IgG subclass involved in the recruitment of C1, an initiator of the classical complement pathway, the lack of IgG3 induction observed in this study may explain results from previous studies (Zaiss *et al.*, 2008). A weak IgG3 response has also been observed against B19V, where virus-specific IgG3 levels are high in early infection but decline significantly over time (Corcoran *et al.*, 2000; Franssila *et al.*, 1996).

AAV-2-specific IgG4 represented less than 10 % of total IgG and levels were variable (2.5±3.8 μg ml^−1^) (Fig. 1e[Fig f1]). In comparison with the serology of other viruses, variable IgG4 levels are perhaps unremarkable. Hepatitis B infection and vaccination typically induce little IgG4, but levels increase post-infection or vaccination. Variable titres of IgG4 are also seen in B19V infection, and typically this is not observed until some 200 days post-infection (Franssila *et al.*, 1996). IgG4 is a feature of the response to HTLV-1 and measles (Lal *et al.*, 1993; Toptygina *et al.*, 2005). If the temporal characteristics of AAV-2 serology mirror those of parvovirus B19V, the minimal IgG3 and elevated IgG4 levels detected here may be representative of late convalescent AAV-2 infections. As IgG3 and IgG4 levels are an indicator of the stage of convalescence in B19V infection (Franssila *et al.*, 1996), further examination of the temporal characteristics of IgG3 and IgG4 induction in AAV-2 infection might be useful.

Few studies have examined human cell-mediated immune responses to AAV-2 (Chirmule *et al.*, 1999; Manno *et al.*, 2006). This study demonstrated that AAV-2 evokes robust proliferative and cytokine recall responses detectable from PBMC cultures. Of the 41 donors examined, 19 demonstrated a statistically significant proliferative response to stimulation with AAV-2 (Fig. 2). Chirmule *et al.* (1999) also examined human PBMC proliferation in response to AAV-2 but found that only 3 of 57 of their subjects produced a stimulation index greater than 2.0. This discrepancy may be due to the relatively low concentration of AAV-2 used for the restimulation in that study (m.o.i. of 100, compared with 10 000 here).

The cytokine profiles evoked by AAV-2 did not exhibit consistent Th1 or Th2 polarization in this study. IFN-*γ* was the most frequently detected cytokine (Fig. 3a[Fig f3]), indicating that, in some subjects, AAV-2 evokes a Th1-like response. IL-13, an indicator of Th2 responses, was only detected from weakly proliferating cultures (SI between 1.5 and 3) (Fig. 3b[Fig f3]) whereas IL-10 production was detected across a range of donors (Fig. 3c[Fig f3]). Chirmule *et al.* (1999) also examined AAV-2-stimulated PBMC cultures for cytokines, finding IFN-*γ* and IL-10 in 6 and 12 % of the cultures, but these authors examined IL-4 instead of IL-13, failing to find the cytokine in any culture.

The AAV-2 capsid is composed of three proteins: VP1, VP2 and VP3 in a ratio of 1 : 1 : 20 (Xie *et al.*, 2002). VP2 and VP3 are products of the splicing of VP1 mRNA and both proteins represent a sub-sequence of the VP1 protein. Using a conservative definition, 17 epitopes were identified as recognized on the capsid of AAV-2 VP1 (Table 2). A further 42 sequences were recognized by a single donor each (Supplementary Table S2). Limitations in assay sensitivity and the conservative epitope definition employed mean that it is likely that more epitopes are recognized than defined in this study.

The panel of epitopes identified herein includes some sequences (Supplementary Table S2) previously identified in human and mouse studies (Chen *et al.*, 2006; Manno *et al.*, 2006; Sabatino *et al.*, 2005). The RDSLVNPGPAMA and EIQYTSNYNKSV sequences recognized by donor 13 were similar to sequences identified in C57BL/6 mice (Sabatino *et al.*, 2005). The sequence GFRPKRLNFKLF recognized by donor 16 shares an 11 aa identity with a 15-mer sequence identified in BALB/c mice (Sabatino *et al.*, 2005). Likewise, the sequence VPQYGYLTL identified as an epitope in BALB/c mice (Sabatino *et al.*, 2005) as well as in a single human case by Manno *et al.* (2006) lies within the sequence VFMVPQYGYLTL identified as a candidate epitope for donor 16. Furthermore, Chen *et al.* (2006) identified an immunogenic sequence TSADNNNSEYSWTGA in mice which spans two sequences recognized by donor 50 (SKTSADNNNSEY and NSEYSWTGATKY).

The panel of 17 epitopes recognized by two or more donors in this study have not been previously identified in human or animal models, with two exceptions. Chen *et al.* (2006) identified the sequence QVSVEIEWELQKENS in mice, and this sequence shares 11 aa with the candidate epitope EIEWELQKENSK (sequence C, Table 2) recognized by three donors (13, 50 and 51) in this study. The second sequence, FKLFNIQV (sequence K, Table 2), was recognized by donors 16 and 50 and is homologous to a sequence identified in mice by Sabatino *et al.* (2005). Sequences B and C were each recognized by three donors, whilst sequence A was recognized by four. One limitation of the approach employed to identify these sequences was the peptide of only 12 residues, a size that would not be optimal for defining class II-restricted epitopes. Furthermore, the definition used here to delineate T-cell epitopes was stringent. Therefore, the present study has probably underestimated the number of T-cell epitopes for AAV-2 recognized by the study population. Despite these limitations, the number of epitopes identified highlights both the prevalence and diversity of T-cell memory for AAV-2 in the study population.

It was not possible to identify a correlation between donor HLA haplotype and the corresponding epitopes recognized in this study. Now that a large number of epitopes of AAV-2 have been identified, it would be valuable in future to use a larger sample size and include approaches to assign particular epitopes to specific HLA alleles. The haplotypes of AAV-2 responding donors did display diversity (Table 3). This was also the case for donors responding to the most common stimulating capsid sequences (Table 4). It is reported here that the DQ2, DQ3 [DQ7(3), DQ8(3), DQ9(3)] and DQ6(1) serotype alleles were present at high frequencies in our study group. These results agree with recent detailed examinations of HLA allele frequencies and haplotypes in the Irish population (Dunne *et al.*, 2008). The observation that a number of AAV-2 sequences can be recognized by donors with different HLA haplotypes, and indeed across species, indicates that the epitopes described in Table 2 might reasonably be considered to be immunodominant human T-cell epitopes.

It is intriguing that recent reports have indicated successful therapeutic use of AAV via the subretinal route (Hauswirth *et al.*, 2008) whilst intravenous and intramuscular administrations have been less successful (Brantly *et al.*, 2006; Manno *et al.*, 2006). The prevalence of humoral and cell-mediated immunological memory for AAV-2 demonstrated here is likely to contribute to the failure of intravenous and intramuscular administration. Such limitations might not apply to antigen encountered via the eye; it is well known that powerful immunosuppressive effects can be induced when antigen is introduced to the ocular anterior chamber leading to the phenomenon of anterior-chamber-associated immune deviation (Streilein, 2003). It may be that a subretinal route for AAV gene therapies induce such phenomena and avoid confounding immunological memory.

It has been suggested that AAV-2 capsid modification enhances the efficacy of the virus as a vector (Monahan & Samulski, 2000) or that immunosuppressive drugs following vector administration prevent cell-mediated immunity (Manno *et al.*, 2006). However, systemic immunosuppression may be undesirable in candidates for gene therapy and thus the immunodominant sequences identified here may thus represent appropriate targets for capsid modification in rare cases. Nevertheless, our observation that even a small sample of an outbred population can support diverse recall response against multiple T-cell epitopes means that genetic modification of AAV-2 to escape immune recognition by T cells fully is not a feasible goal. Vector capsid modification to remove identified epitopes might instead represent a strategy reserved for specific cases in which administration of immunosuppressive drugs is undesirable. Serology did not reliably predict the quality of T-cell memory for AAV-2 (Table 1). Thus, detailed combined screening of patient antibody and T-cell epitope recognition would be valuable tools in such cases.

## Supplementary Material

[Supplementary Material]

## Figures and Tables

**Table 1. t1:** Cytokine and serological profiles of cultures responding to AAV-2

**Donor**	AAV-2-specific IgG subclass (% total IgG)*				**PBMC proliferation (SI)**	**Cytokine increase (pg ml^−1^)†**		
	**1**	**2**	**3**	**4**		**IFN-*γ***	**IL-10**	**IL-13**
**19**	39	59	2	0	1.75	−	−	−
**46**	48	39	3	9	2.08	−	−	189
**45**	60	30	3	7	2.20	−	209	−
**16**	48	49	3	0	2.22	−	−	−
**27**	48	41	3	8	2.27	5	−	1633
**31**	90	0	1	8	2.31	7	67	1281
**32**	32	64	3	0	2.33	3	−	962
**47**	57	21	4	18	2.39	−	225	−
**13**	37	53	4	5	2.57	85	−	−
**43**	33	49	2	15	3.29	−	126	−
**51**	37	62	2	0	5.10	221	138	−
**38**	43	37	4	16	6.25	39	91	−
**50**	29	48	2	20	10.91	546	141	−
**40**	59	25	4	11	14.77	49	132	−
**15**	44	49	3	3	18.65	110	−	−
**41**	22	75	1	2	25.50	532	−	−

*AAV-2-specific IgG subclass levels were determined by indirect ELISA. Detectable specific subclass levels are expressed as a percentage of the total specific IgG. †Cytokine levels in proliferation assay supernatants were determined by sandwich ELISA. Intermediate and negative cytokine production levels are indicated (−). Increases, where applicable, are measured in pg ml^−1^.

**Table 2. t2:** Common VP1 consensus sequences recognized by human PBMCs

Peptide*	**VP1 consensus sequence†**	**Responding donors**	**SYFPEITHI prediction**		**Epitope**
			**Class I score‡**	**Class II score§**	
321–333	KEVTQNDGTTTI	16, 19, 50, 51	24	20	A
241–253	TTSTRTWALPTY	16, 50, 51	21	24	B
681–693	EIEWELQKENSK	13, 50, 51	21	26	C
9–21	DWLEDTLSEGIR	16, 50	21	32	D
57–69	NGLDKGEPVNEA	16, 50	20	27	E
113–125	NLGRAVFQAKKR	50, 51	28	20	F
121–133	AKKRVLEPLGLV	40, 50	29	20	G
249–261	LPTYNNHLYKQI	16, 51	24	24	H
257–269	YKQISSQSGASN	16, 51	–	23	I
265–277	GASNDNHYFGYS	16, 51	26	–	J
313–325	FKLFNIQVKEVT	16, 50	–	23	K
329–341	TTTIANNLTSTV	19, 50	22	24	L
393–405	YCLEYFPSQMLR	16, 50	21	28	M
457–469	QSRLQFSQAGAS	13, 16	–	25	N
553–565	DIEKVMITDEEE	13, 50	21	30	O
716–728	TNGVYSEPRPIGTRYLT	16, 51	24	21	P
505–516	ATKYHLNGRDSL	13, 50, 51	21	25	Q

*Peptide number corresponds to amino acid sequence of VP1. The full lists of peptides and sequences are detailed in Supplementary Tables S1 and S2. †Consensus sequences were derived from the 12-mer overlap between pairs of PBMC-stimulating VP1 peptides with the exception of the overlap between peptides 90 and 91 which was of 17 amino acids. Pairs of peptide were considered positive if both produced an SI value >1 sd above the mean SI. ‡VP1 sequence was analysed using SYFPEITHI prediction for alleles of the HLA-A and HLA-B loci; output sequences were nonamers. Scores represent an arbitrary peptide binding capacity for each peptide. §VP1 sequence was analysed using SYFPEITHI prediction for alleles of the HLA-DRB locus; output sequences were 15-mers. For both class I and II, the best score obtained is reported. Scores below 20 are not reported.

**Table 3. t3:** HLA profile of donor PBMCs responding to AAV-2

**Donor**	Class I HLA allele*	Class II HLA allele*
**13**	A2, B7, B13, Cw5, Cw7	DR4/DR53, DR7/DR53, DQ2, DQ8(3)
**15**	B7, B8, Cw7	DR17(3)/DR52, DR15(2)/DR51, DQ2, DQ6(1)
**16**	A1, Cw7, Cw7	DR17(3)/DR52, DR51, DQ2, DQ6(1)
**19**	A68, B8, Cw7, Cw8	DR3/DR52, DR17(3)/DR52, DQ2, DQ6(1)
**27**	A1, A2, B*4440, B*5615, Cw5, Cw7	DQ2, DQ7(3)
**31**	A1, B8, B51(5)	DR17(3)/DR52, DR8, DQ2, DQ4
**32**	A1, A29(19), Cw7, Cw7	DR17(3)/DR52, –/DR52, DQ2, DQ6(1)
**38**	A1, B63(15), B44(12), Cw5, Cw7	DR4/DR53, DR13(6)/DR52, DQ7(3), DQ6(1)
**40**	Cw1, Cw7	DR1, DR7/DR53, DQ2, DQ5(1)
**41**	A2, B44(12), B47, Cw4	DR4/DR53, DR7/DR53, DQ2, DQ7(3)
**43**	A31(19), B7, B71(70), Cw18, Cw6, Cw18	DR4/DR53, DR15(2)/DR51, DQ6(1), DQ6(1)
**45**	A2, A24(9), B7, B18, Cw5, Cw7	DR17(3)/DR52, DR15(2)/DR51, DQ2, DQ6(1)
**46**	B55(2), Cw1, Cw4	DR103, DR13(6)/DR52, DQ5(1), DQ6(1)
**47**	A1, B42	DR4/DR53, DR4/DR53, DQ8(3), DQ5(1)
**50**	A31(9), B*14, B*14, Cw*06, Cw*08	DR13(6)/DR52, DR15(2)/DR51, DQ9(3)
**51**	A66(10)/A26(10), B44(12), B57(17), Cw5, Cw6	DR4/DR53, DR15(2)/DR51, DQ6(1), DQ6(1)

*HLA type I and II alleles were determined by SSP PCR. Alleles omitted where not determined.

**Table 4. t4:** Donor PBMCs with diverse HLA haplotypes recognize common consensus sequences of AAV nd, Not determined.

**Donor**	HLA class I*			HLA class II*		**Epitopes recognized**
	**A**	**B**	**C**	**DR**	**DQ**	
**13**	nd	nd	Cw1, Cw7	DR1, DR7/DR53	DQ2, DQ5(1)	C, N, O, Q
**16**	A68	B8	Cw7, Cw8	DR3/DR52, DR17(3)/DR52	DQ2, DQ6(1)	A, B, D, E, H, I, J, K, M, N, P
**19**	A2	B7, B13	Cw5, Cw7	DR4/DR53, DR7/DR53	DQ2, DQ8(3)	L
**40**	A1	B42	nd	DR4/DR53, DR4/DR53	DQ8(3), DQ5(1)	G
**50**	nd	B55(2)	Cw1, Cw4	DR103, DR13(6)/DR52	DQ5(1), DQ6(1)	A, B, C, D, E, F, G, K, L, M, O, Q
**51**	A31(19)	B7, B71(70)	Cw18/Cw6, Cw18	DR4/DR53, DR15(2)/DR51	DQ6(1), DQ6(1)	A, B, C, F, H, I, J, P, Q

*HLA class I and II alleles were determined by SSP PCR.
