# Supplementary material for: Adeno-associated virus serotype 2 induces cell-mediated immune responses directed against multiple epitopes of the capsid protein VP1
Source: J Gen Virol. 2009 Nov;90(Pt 11):2622–33. doi: 10.1099/vir.0.014175-0 (PMC2885037; doi:10.1099/vir.0.014175-0)
Supplement: [Supplementary Material] [file supp_90_11_2622__index.html]

 Adeno-associated virus serotype 2 induces cell-mediated immune responses directed against multiple epitopes of the capsid protein VP1 -- Madsen et al. 90 (11): 2622 Data Supplement - Supplementary Material -- Journal of General Virology

### Adeno-associated virus serotype 2 induces cell-mediated immune responses directed against multiple epitopes of the capsid protein VP1, by D. Madsen, E. R. Cantwell, T. O'Brien, P. A. Johnson and B. P. Mahon

*Journal of General Virology* vol. **90**, part 11, pp. 2622 - 2633

**Supplementary Fig. S1.** Verification of the capacity of the IgG subclass ELISA protocol to successfully detect antigen-bound IgG3. [PDF] (136 KB)

**Supplementary Table S1.** Sequences of the 20-mer peptides derived from the AAV-2 VP1 capsid protein

**Supplementary Table S2.** AAV-2 VP1 capsid sequences recognized by human PBMC.

[PDF of tables] (47 KB)

  
  
