# Supplementary material for: Adeno-associated virus serotype 2 induces cell-mediated immune responses directed against multiple epitopes of the capsid protein VP1
Source: J Gen Virol. 2009 Nov;90(Pt 11):2622–33. doi: 10.1099/vir.0.014175-0 (PMC2885037; doi:10.1099/vir.0.014175-0)
Supplement: [Supplementary Material] [file supp_90_11_2622__1.pdf]

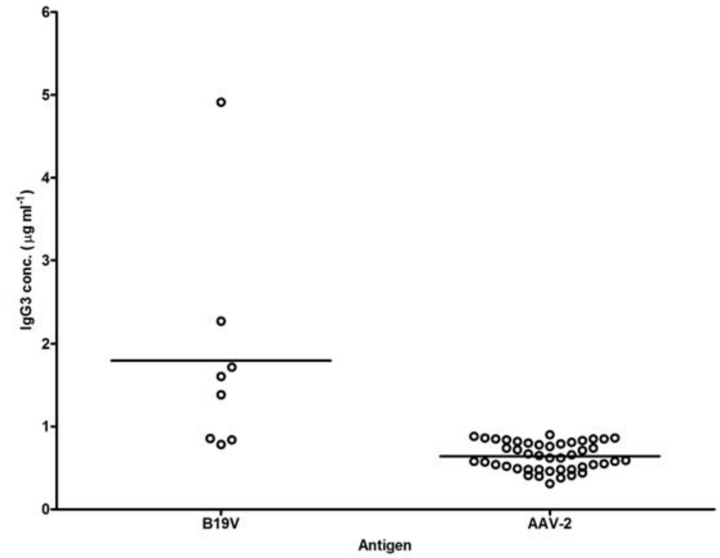

Supplementary Fig. S1. Verification of the capacity of the IgG subclass ELISA protocol to successfully detect antigen-bound IgG3. AAV-2 specific IgG3 levels determined in plasma from healthy humans (*n* = 41) are as previously shown (Fig. 2c of the main paper). IgG3 specific for the VP2 capsid protein (2 μg ml^{-1}) of Parvovirus B19 (B19V) was detected in sera from known B19V-positive humans (*n* = 8) using the same detection reagents as used in Fig. 2(c) of the main paper.

Madsen, D., Cantwell, E. R., O'Brien, T., Johnson, P. A. & Mahon, B. P. (2009). Adeno-associated virus serotype 2 induces cell-mediated immune responses directed against multiple epitopes of the capsid protein VP1. *J Gen Virol* **90**, 2622–2633.
