# Supplementary material for: Adeno-associated virus serotype 2 induces cell-mediated immune responses directed against multiple epitopes of the capsid protein VP1
Source: J Gen Virol. 2009 Nov;90(Pt 11):2622–33. doi: 10.1099/vir.0.014175-0 (PMC2885037; doi:10.1099/vir.0.014175-0)
Supplement: [Supplementary Material] [file supp_90_11_2622__2.pdf]

Supplementary Table S1. Sequences of the 20-mer peptides derived from the AAV-2 VP1 capsid protein

Each VP1 peptide was synthesized with a 12-mer consensus overlap with adjacent peptides. Peptides 90 and 91 had a 17-mer overlap.

Donor	Sequence	Donor	Sequence
**1**	MAADGYLPDWLEDTLSEGIR	**23**	GDADSVPDPQPLGQPPAAPS
**2**	DWLEDTLSEGIRQWWKLKPG	**24**	PQPLGQPPAAPSGLGTNTMA
**3**	EGIRQWWKLKPGPPPPKPAE	**25**	AAPSGLGTNTMATGSGAPMA
**4**	LKPGPPPPKPAERHKDDSRG	**26**	NTMATGSGAPMADNNEGADG
**5**	KPAERHKDDSRGLVLPGYKY	**27**	APMADNNEGADGVGNSSGNW
**6**	DSRGLVLPGYKYLGPFNGLD	**28**	GADGVGNSSGNWHCDSTWMG
**7**	GYKYLGPFNGLDKGEPVNEA	**29**	SGNWHCDSTWMGDRVITTST
**8**	NGLDKGEPVNEADAAALEHD	**30**	TWMGDRVITTSTRTWALPTY
**9**	VNEADAAALEHDKAYDRQLD	**31**	TTSTRTWALPTYNNHLYKQI
**10**	LEHDKAYDRQLDSGDNPYLK	**32**	LPTYNNHLYKQISSQSGASN
**11**	RQLDSGDNPYLKYNHADAEF	**33**	YKQISSQSGASNDNHYFGYS
**12**	PYLKYNHADAEFQERLKEDT	**34**	GASNDNHYFGYSTPWGYFDF
**13**	DAEFQERLKEDTSFGGNLGR	**35**	FGYSTPWGYFDFNRFHCHFS
**14**	KEDTSFGGNLGRAVFQAKKR	**36**	YFDFNRFHCHFSPRDWQRLI
**15**	NLGRAVFQAKKRVLEPLGLV	**37**	CHFSPRDWQRLINNNWGFRP
**16**	AKKRVLEPLGLVEEPVKTAP	**38**	QRLINNNWGFRPKRLNFKLF
**17**	LGLVEEPVKTAPGKKRPVEH	**39**	GFRPKRLNFKLFNIQVKEVT
**18**	KTAPGKKRPVEHSPVEPDSS	**40**	FKLFNIQVKEVTQNDGTTTI
**19**	PVEHSPVEPDSSSGTGKAGQ	**41**	KEVTQNDGTTTIANNLTSTV
**20**	PDSSSGTGKAGQQPARKRLN	**42**	TTTIANNLTSTVQVFTDSEY
**21**	KAGQQPARKRLNFGQTGDAD	**43**	TSTVQVFTDSEYQLPYVLGS
**22**	KRLNFGQTGDADSVPDPQPL	**44**	DSEYQLPYVLGSAHQGCLPP

Madsen, D., Cantwell, E. R., O'Brien, T., Johnson, P. A. & Mahon, B. P. (2009). Adeno-associated virus serotype 2 induces cell-mediated immune responses directed against multiple epitopes of the capsid protein VP1. *J Gen Virol* **90**, 2622–2633.

Donor	Sequence	Donor	Sequence
**45**	VLGSAHQGCLPPFPADVFMV	**70**	DIEKVMITDEEEIRTTNPVA
**46**	CLPPFPADVFMVPQYGYLTL	**71**	DEEEIRTTNPVATEQYGSVS
**47**	VFMVPQYGYLTLNNGSQAVG	**72**	NPVATEQYGSVSTNLQRGNR
**48**	YLTLNNGSQAVGRSSFYCLE	**73**	GSVSTNLQRGNRQAATADVN
**49**	QAVGRSSFYCLEYFPSQMLR	**74**	RGNRQAATADVNTQGVLPGM
**50**	YCLEYFPSQMLRTGNNFTFS	**75**	ADVNTQGVLPGMVWQDRDVY
**51**	QMLRTGNNFTFSYTFEDVPF	**76**	LPGMVWQDRDVYLQGPIWAK
**52**	FTFSYTFEDVPFHSSYAHSQ	**77**	RDVYLQGPIWAKIPHTDGHF
**53**	DVPFHSSYAHSQSLDRLMNP	**78**	IWAKIPHTDGHFHPSPLMGG
**54**	AHSQSLDRLMNPLIDQYLYY	**79**	DGHFHPSPLMGGFGLKHPPP
**55**	LMNPLIDQYLYYLSRTNTPS	**80**	LMGGFGLKHPPPQILIKNTP
**56**	YLYYLSRTNTPSGTTTQSRL	**81**	HPPPQILIKNTPVPANPSTT
**57**	NTPSGTTTQSRLQFSQAGAS	**82**	KNTPVPANPSTTFSAAKFAS
**58**	QSRLQFSQAGASDIRDQSRN	**83**	PSTTFSAAKFASFITQYSTG
**59**	AGASDIRDQSRNWLPGPCYR	**84**	KFASFITQYSTGQVSVEIEW
**60**	QSRNWLPGPCYRQQRVSKTS	**85**	YSTGQVSVEIEWELQKENSK
**61**	PCYRQQRVSKTSADNNNSEY	**86**	EIEWELQKENSKRWNPEIQY
**62**	SKTSADNNNSEYSWTGATKY	**87**	ENSKRWNPEIQYTSNYNKSV
**63**	NSEYSWTGATKYHLNGRDSL	**88**	EIQYTSNYNKSVNVDFTVDT
**64**	ATKYHLNGRDSLVNPGPAMA	**89**	NKSVNVDFTVDTNGVYSEPR
**65**	RDSLVNPGPAMASHKDDEEK	**90**	TVDTNGVYSEPRPIGTRYLT
**66**	PAMASHKDDEEKFFPQSGVL	**91**	TNGVYSEPRPIGTRYLTRNL
**67**	DEEKFFPQSGVLIFGKQGSE		
**68**	SGVLIFGKQGSEKTNVDIEK		
**69**	QGSEKTNVDIEKVMITDEEE		

Madsen, D., Cantwell, E. R., O'Brien, T., Johnson, P. A. & Mahon, B. P. (2009). Adeno-associated virus serotype 2 induces cell-mediated immune responses directed against multiple epitopes of the capsid protein VP1. *J Gen Virol* **90**, 2622–2633.

Supplementary Table S2. AAV-2 VP1 capsid sequences recognized by human PBMC

No proliferative response was detected for the following peptides (omitted from the table): 5, 6, 11, 12, 18–21, 23, 24, 26–28, 34, 35, 42–45, 51–53, 66, 68, 71–73, 76, 77, 81, 82 and 89.

**Peptide**	**Donor**	**Sequence**	**Peptide**	**Donor**	**Sequence**
**1**	16, 50	DWLEDTLSEGIR	**37**	16	QRLINNNWGFRP
**2**	50	EGIRQWWKLKPG	**38**	16	GFRPKRLNFKLF
**3**	50	LKPGPPPPKPAE	**39**	16, 50	FKLFNIQVKEVT
**4**	50	KPAERHKDDSRG	**40**	16, 19, 50, 51	KEVTQNDGTTTI
**7**	16, 50	NGLDKGEPVNEA	**41**	19, 50	TTTIANNLTSTV
**8**	50	VNEADAAALEHD	**46**	16	CLPPFPADVFMV
**9**	50	LEHDKAYDRQLD	**47**	16	VFMVPQYGYLTL
**10**	50	RQLDSGDNPYLK	**48**	50	YLTLNNGSQAVG
**13**	50	KEDTSFGGNLGR	**49**	50	QAVGRSSFYCLE
**14**	50, 51	NLGRAVFQAKKR	**50**	16, 50	YCLEYFPSQMLR
**15**	40, 50	AKKRVLEPLGLV	**54**	16	AHSQSLDRLMNP
**16**	50	LGLVEEPVKTAP	**55**	16	LMNPLIDQYLYY
**17**	50	KTAPGKKRPVEH	**56**	13	YLYYLSRTNTPS
**22**	16	GDADSVPDPQPL	**57**	13	NTPSGTTTQSRL
**25**	51	NTMATGSGAPMA	**58**	13, 16	QSRLQFSQAGAS
**29**	50	TWMGDRVITTST	**59**	16	AGASDIRDQSRN
**30**	16, 50, 51	TTSTRTWALPTY	**60**	50	QSRNWLPGPCYR
**31**	16, 51	LPTYNNHLYKQI	**61**	50	PCYRQQRVSKTS
**32**	16, 51	YKQISSQSGASN	**62**	50	SKTSADNNNSEY
**33**	16, 51	GASNDNHYFGYS	**63**	50	NSEYSWTGATKY
**36**	16	CHFSPRDWQRLI	64	13, 50, 51	ATKYHLNGRDSL

Madsen, D., Cantwell, E. R., O'Brien, T., Johnson, P. A. & Mahon, B. P. (2009). Adeno-associated virus serotype 2 induces cell-mediated immune responses directed against multiple epitopes of the capsid protein VP1. *J Gen Virol* **90**, 2622–2633.

Peptide	Donor	Sequence	Peptide	Donor	Sequence
**65**	13	RDSLVNPGPAMA	**83**	16	PSTTFSAAKFAS
**67**	51	DEEKFFPQSGVL	**84**	51	KFASFITQYSTG
**69**	50	QGSEKTNVDIEK	**85**	51	YSTGQVSVEIEW
**70**	13, 50	DIEKVMITDEEE	**86**	13, 50, 51	EIEWELQKENSK
**74**	16	RGNRQAATADVN	**87**	13	ENSKRWNPEIQY
**75**	13	ADVNTQGVLPGM	**88**	13	EIQYTSNYNKSV
**78**	13	IWAKIPHTDGHF	**90**	51	TVDTNGVYSEPR
**79**	13	DGHFHPSPLMGG	**91**	16, 51	TNGVYSEPRPIGTRYLT
**80**	13	LMGGFGLKHPPP			

Madsen, D., Cantwell, E. R., O'Brien, T., Johnson, P. A. & Mahon, B. P. (2009). Adeno-associated virus serotype 2 induces cell-mediated immune responses directed against multiple epitopes of the capsid protein VP1. *J Gen Virol* **90**, 2622–2633.
